# Supplementary material for: The Relationship between Iron and LRRK2 in a 6-OHDA-Induced Parkinson’s Disease Model
Source: Int J Mol Sci. 2023 Feb 13;24(4):3709. doi: 10.3390/ijms24043709 (PMC9964371; doi:10.3390/ijms24043709)
Supplement: Supplementary file 1 [file ijms-24-03709-s001.zip › ijms-2123972-supplementary.pdf]

Supplementary Information for

The relationship between iron and LRRK2 in 6-OHDA-induced Parkinson's disease model

Ruru Jia[1#], Yanling Liu[1#], Ke Shuai[1], Cheng Zhou[1], Lei Chen[1], Li Zhu[1,2*], Xiao-Mei Wu[1,2*]

This includes Supplementary text and Figures S1.

1. *6-OHDA and Ferric ammonium citrate decrease the viability of SH-SY5Y cells*

SH-SY5Y cells were treated with the different concentrations of 6-OHDA or ferric ammonium citrate (FAC) for 24 h. Then, the cell viability was detected by MTT assay. It was found that 6-OHDA induced the decrease of cell viability to 90% of control at 25 µM, 79% at 50 µM and 61% at 100 µM. FAC showed the similar trend to that of 6-OHDA. The cell viability also was gradually decreased with increase in FAC concentration, reached about 86% of control at 2.5 µg/ml, 69% at 5 µg/ml and 49% at 10 µg/ml. These data suggest that 6-OHDA and FAC both can cause the damage of SH-SY5Y cells in a dose dependent manner, 100 µM of 6-OHDA and 5 µg/ml of FAC are chosen to use in subsequent experiments.

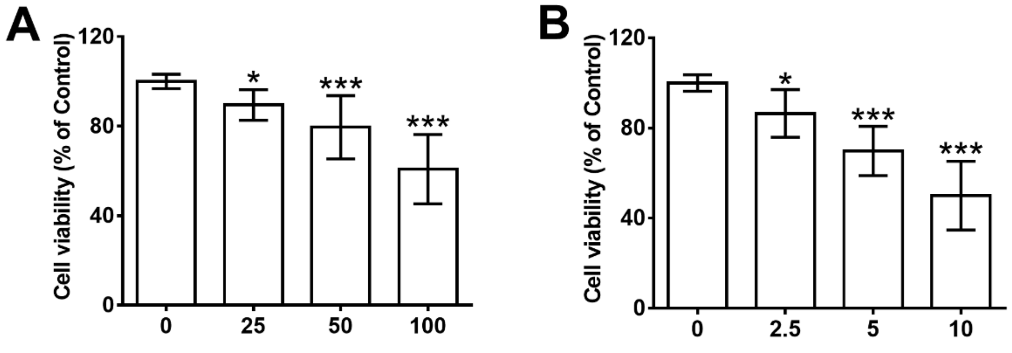

Supplementary Figure S1. 6-OHDA and FAC decreases the viability of SH-SY5Y cells. SH-SY5Y cells were treated with 6-OHDA (0, 25, 50, 100 µM) or FAC (2.5, 5, 10 µg/ml) for 24 h. (A) Cell viability after 6-OHDA treatment. (B) Cell viability after FAC treatment. *P < 0.05, ***P < 0.001 (n = 10).
